# Metastasizing Ameloblastoma Mimicking Squamous Cell Carcinoma of the Lung and Harboring an AKT1 Mutation

**DOI:** 10.1007/s12105-025-01844-5

**Published:** 2025-10-27

**Authors:** James Arrich, Steven Forrest, Matthew Lofthouse, Asterios Triantafyllou, Keith D. Hunter, Alexander Haragan

**Affiliations:** 1 Cellular Pathology, Liverpool Clinical Laboratories, University Hospitals of Liverpool Group, Liverpool, UK; 2 Liverpool Head and Neck Center, Molecular and Clinical Cancer Medicine, University of Liverpool, 6 West Derby Street, Liverpool, L7 8TX UK

**Keywords:** Metastasizing ameloblastoma, Odontogenic tumor, Neoadjuvant therapy, Lung, Squamous cell carcinoma, AKT1

## Abstract

We present a case of a 41-year-old non-smoker male patient presenting with ameloblastoma which metastasized to the lung 14 years post excision of the primary mandibular tumor. A biopsy of the pulmonary mass was initially diagnosed as squamous cell carcinoma by a non-specialist pathologist who was not provided a history of the patient’s prior ameloblastoma diagnosis. The patient subsequently underwent neoadjuvant chemo-immunotherapy followed by surgical resection of the lung mass. The definitive diagnosis of metastasizing ameloblastoma (MA) was made on the surgical resection and compared to the original mandible lesion. Confounding morphological features were attributed to the impact of neoadjuvant treatment. Molecular analysis of the metastasis and original jaw lesion revealed a mutation of *AKT1* (c.49G > A p.(Glu17Lys) COSMIC ID: COSM33765) but no mutations in the *BRAF* gene were identified. This case illustrates the critical necessity of a thorough clinical history of previous neoplasia, even many years following treatment, as well as the role of molecular profiling for identifying novel targeted treatment options.

**Supplementary Information:**

The online version contains supplementary material available at 10.1007/s12105-025-01844-5.

## Case Report

### Presentation, Diagnosis and Management

**Fig. 1:**
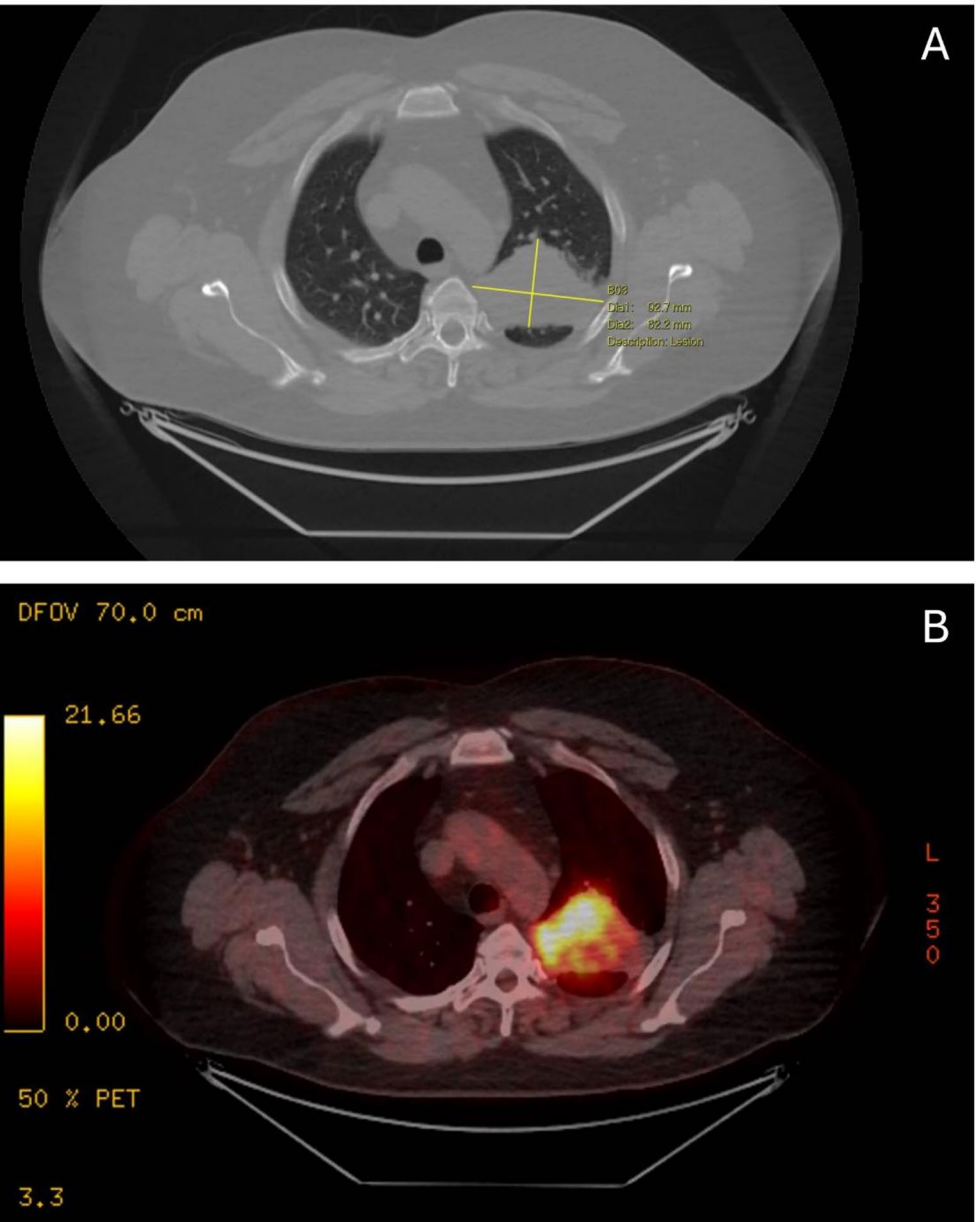
Sectional imaging of the lung lesion on CT **A** and PET CT **B**, demonstrating a large PET avid lesion in the apex of the right lung

A 41-year-old male with a past medical history of type 2 diabetes and previously resected ameloblastoma in the mandible presented to his local hospital with hemoptysis and was found on plain radiography to have a large mass in the apex of his right lung. Further imaging on CT demonstrated a 92.7 mm mass that was highly PET avid. (Fig. 1A and B).

**Fig. 2:**
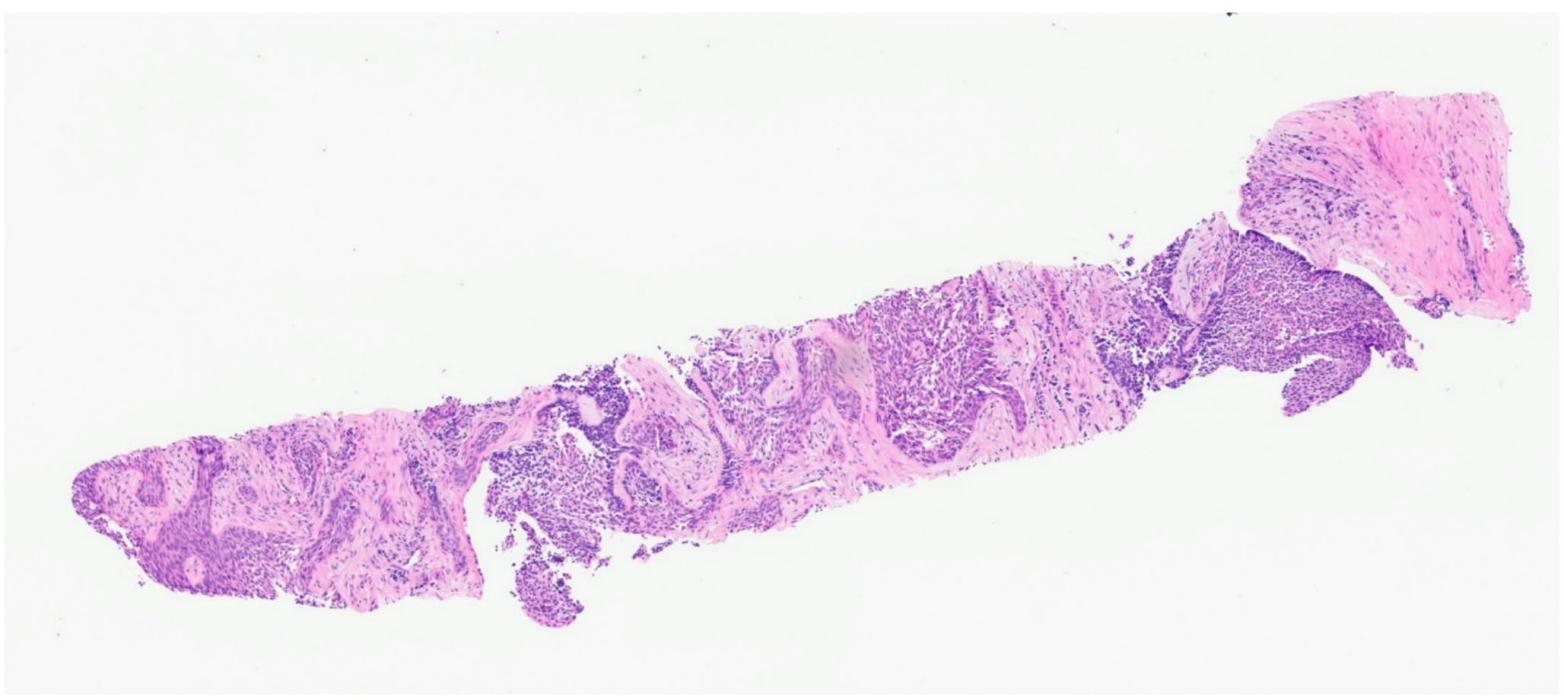
Histological image of the H&E-stained core biopsy of the lung lesion

A core biopsy of this mass was performed at a district general hospital and reported locally as containing cores of fibrotic inflamed tissue infiltrated by islands of tumor cells with plentiful eosinophilic cytoplasm and nuclear pleomorphism (Fig. 2). Immunohistochemistry demonstrated nuclear staining for p40, widespread membranous staining for CD56 and focal cytoplasmic expression for synaptophysin. The tumor cells were also negative for CK7 and TTF-1. A diagnosis of squamous cell carcinoma with focal neuroendocrine differentiation was thus rendered. The patient was then referred to the regional specialist thoracic center for treatment.

The patient was staged radiologically as cT4 cN1 cM0, stage 3 A. Molecular profiling via next generation sequencing, using the Oncomine Precision Assay (Thermo Fisher Scientific), for a standard lung carcinoma panel found no actionable oncogenic drivers in the Standard Lung carcinoma sub-panel (DNA targets: BRAF, EGFR, KRAS & MET exon 14 skipping; RNA fusions: ALK, ROS1, RET, MET, NTRK1, NTRK2 & NTRK3). PD-L1 expression was 1% (Tumor Proportion Score). Following discussion at multidisciplinary team meeting the patient commenced neoadjuvant chemoimmunotherapy regime of combination nivolumab, carboplatin and gemcitabine for three cycles and after successful completion proceeded to surgical resection of the left lung.

**Fig. 3:**
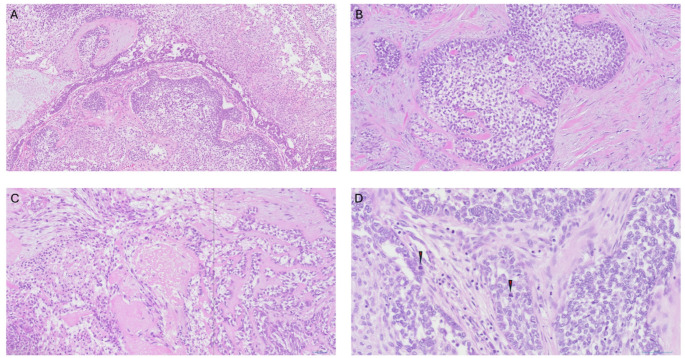
Lung excision histology, H&E-stained sections. **A** x10 and **B** x20, demonstrating islands of epithelial cells with focal peripheral palisading, and central stellate-reticulum-like areas, **C** x20 focal area of necrosis. **D** x40: increased mitotic activity, with mitoses marked

**Fig. 4:**
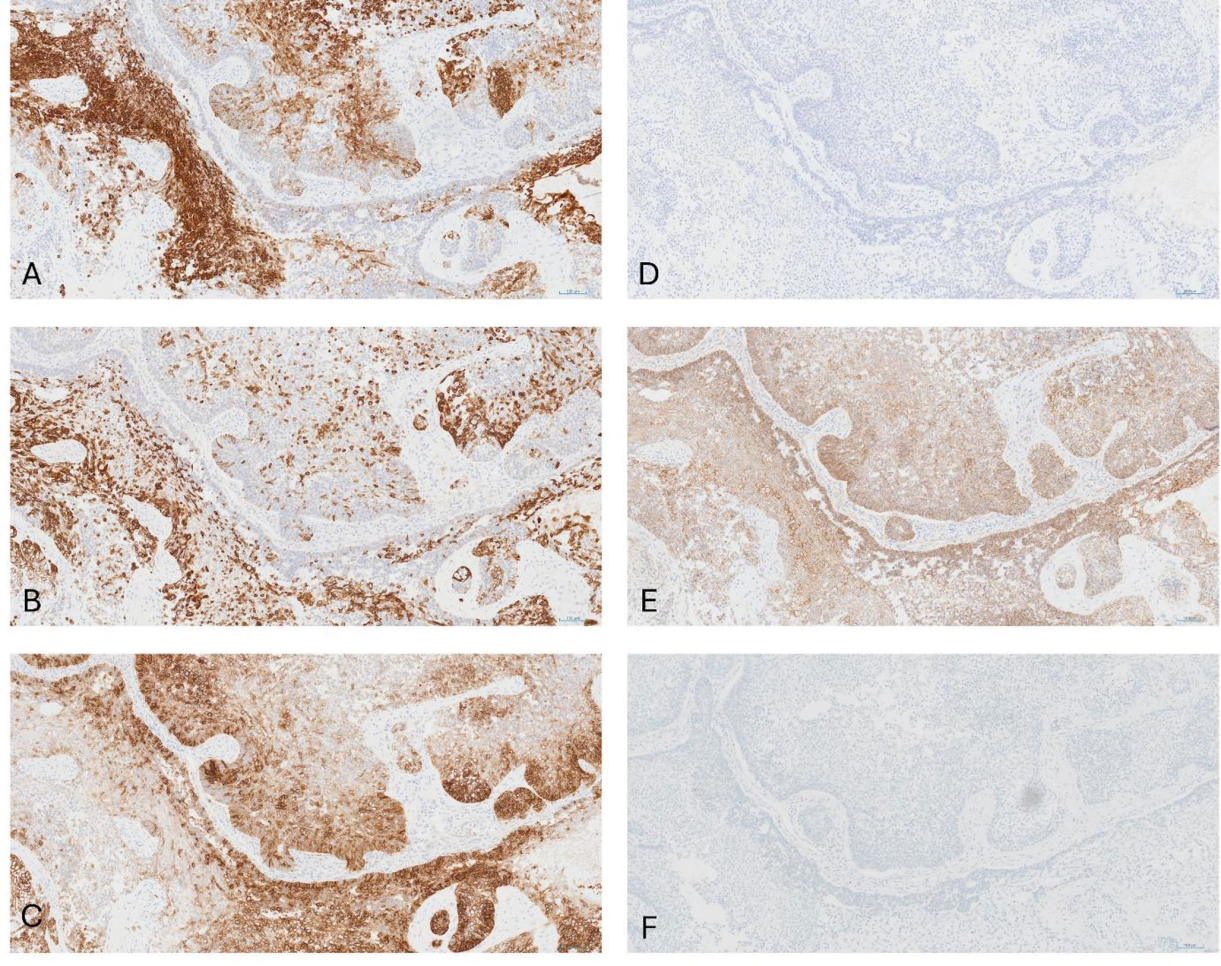
Immuno-histochemical staining for various biomarkers in the lung excision histology. **A** Cytokeratin 19, **B** Cytokeratin 14, **C** CD56, **D** Calretinin, **E** β-Catenin, **F** BRAF V600E

Macroscopic assessment of the left pneumonectomy specimen revealed a dominant mass measuring 65 mm maximally alongside a separate 20 mm intraparenchymal nodule and a 60 mm subpleural nodule. Microscopic assessment found all three masses to have a similar morphology: composed of tumor cells arranged in cords, islands and sheets exhibiting peripheral palisading of a basal cell population surrounding central stellate reticulum like areas with foci of squamous metaplasia (Fig. 3A and B). There was minimal evidence of histological response to neoadjuvant therapy. Within the masses there were multiple small foci of necrosis and an increased mitotic rate but no high-grade cytological morphology was noted (Fig. 3c and D). Immunohistochemical analysis of the tumor cells demonstrated diffuse positive staining for CK19, CK14 and CD56. Beta-catenin showed membranous staining and calretinin and BRAF V600E were negative (Fig. 4). Ki67 positivity was 20–30% in the peripheral basal cell population but < 5% in the central areas. Following discussion between thoracic and head and neck specialist pathologists, a final diagnosis of metastasizing ameloblastoma (MA) was given.

A broader panel of next generation sequencing was performed using the full Oncomine Precision Assay panel (Thermo Fisher Scientific) and GX5 - Solid Tumor – DNA and Fusions – w.3.2.0 workflow (see Supplementary file S1 for full list of analyses) and was found to harbor a AKT1 c.49G > A p.(Glu17Lys) point mutation of exon 4 (COSMIC ID: COSM33765). No other mutations, including BRAF, were identified. The patient is now under the care of the specialist head and neck and thoracic teams.

### Primary Lesion Clinical History

**Fig. 5:**
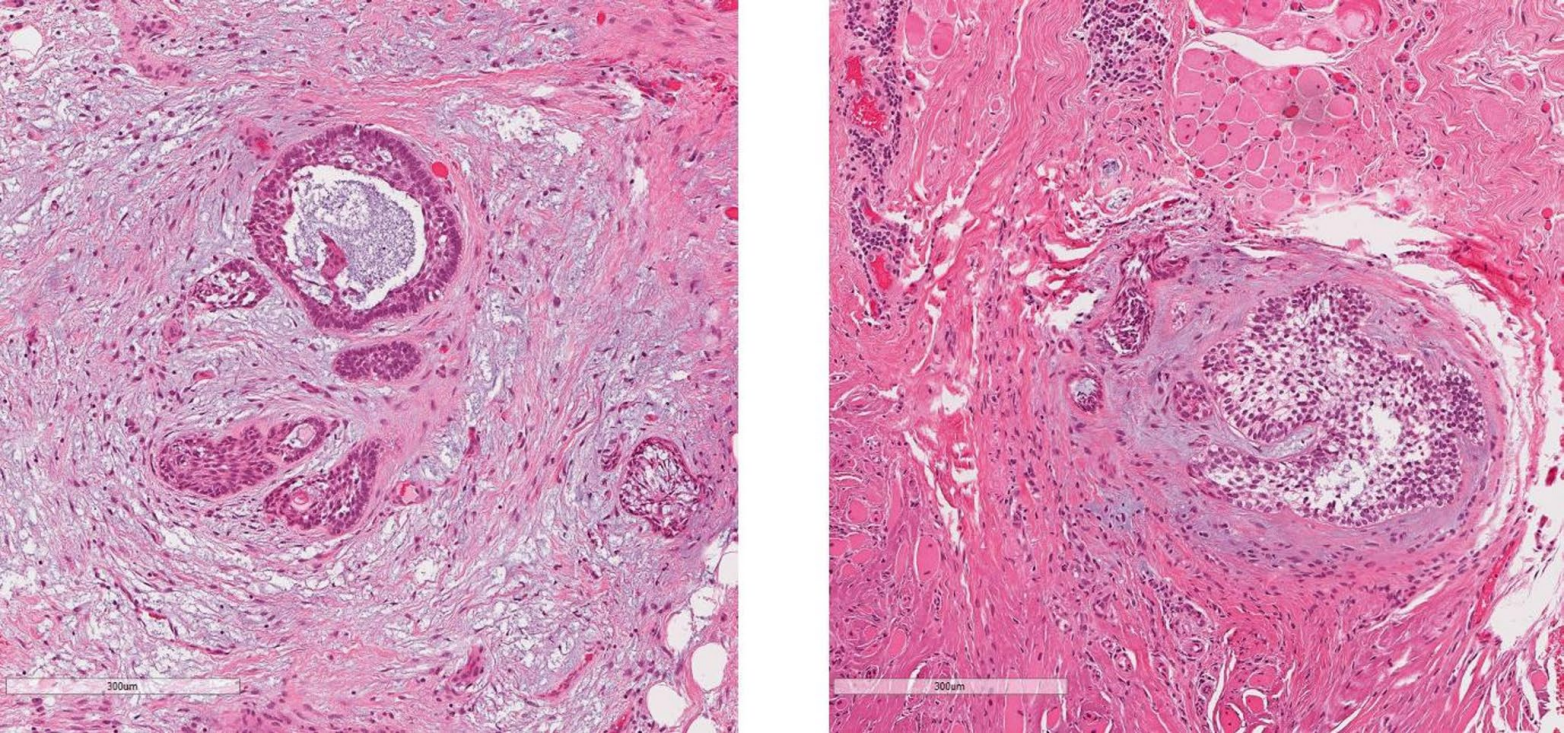
H&E-stained sections from the 2010 recurrence of ameloblastoma in the right posterior mandible

The patient was first treated for a tumor of the right posterior body/ramus of the mandible 14 years prior to the current presentation. The tumor was histologically diagnosed as a conventional solid/multicystic ameloblastoma with a mixed, plexiform and follicular, pattern and focal acanthomatous change by a specialist Oral and Maxillofacial Pathologist. The tumor was treated by surgical resection including initial enucleation of cystic lesions associated with lower right third molar tooth, followed by revision surgery, which included a segmental mandibulectomy. Over a period of five years the patient underwent six procedures for management of the ameloblastoma. At no point were confirmed clear surgical margins obtained. The patient was followed up by yearly radiographic investigation of the reconstructed primary site from 2010, and a small focus of recurrent ameloblastoma was identified at this point (Fig. 5). This was treated by conservative surgical excision. No subsequent evidence of recurrence was detected on yearly follow-up and the patient was discharged from the Oral and Maxillofacial Surgery service in 2016, eight years prior to the current presentation. No evidence of recurrence at the primary site has been identified as part of the work-up following the identification of the lung metastasis.

Earlier histological material was not available, but molecular analysis of the 2010 sample by similar techniques has identified the same AKT1 c.49G > A p.(Glu17Lys) point mutation of exon 4 (COSMIC ID: COSM33765), on a BRAF wild type background.

## Discussion

Ameloblastomas are benign but locally destructive odontogenic tumors. They are also known to spread to distant sites very rarely, despite remaining histologically benign, a phenomenon referred to as metastasizing ameloblastoma (MA). MA are classified as benign odontogenic tumors in the 4th and 5th editions of the WHO Classification of Head and Neck Tumors, having previously been classified as a malignant tumor in previous editions [1]. This contrasts with ameloblastic carcinoma, which is a malignant odontogenic tumor that resembles ameloblastoma to some extent, but is cytologically malignant, and has metastatic potential [1]. The combined annual incidence of metastasizing ameloblastoma and ameloblastic carcinoma has been reported as 1.79 per 10-million-person years, but the historical literature does not make a clear distinction between these entities [2, 3], with both diagnoses included in “malignant ameloblastoma”, making it difficult to determine the true prevalence of metastasizing ameloblastoma. Despite benign morphological features, metastasizing ameloblastoma has been associated with poor prognosis in some series, but less so than ameloblastic carcinoma [4, 5]. Translating these into data for disease specific survival is difficult, but over several overlapping cohorts 11/20 (55%), 12/25 (48%), 11/43 (25%) have been reported as dying with or of MA, with a mean overall survival most commonly reported as being around 5 years [4–7].

Several surveys of the literature have been published, which have aimed at collating the numerous case reports [4–6, 8, 9]. These are of varying quality, and some of the systematic reviews in this area are unhelpful, as due care has not been taken in the assessment of the literature [3]. The more recent ones have attempted to apply stringent criteria for acceptance of cases as MA, as in many case reports it has not been possible to confirm the diagnosis of cytologically benign ameloblastoma in both the primary lesion and metastasis. These criteria, implemented in the paper by van Dam et al., and used by others, such as the paper by Chrcanovic et al., have markedly reduced the number of reported cases (by over half), but these carefully curated cohorts likely give a more accurate representation of the clinical features of MA [4, 6].

Overall, the current case presents several features commonly associated with MA. The initial patient age at diagnosis of the primary lesion varies from 30 to 34 years (27 in this case), with numerous recurrences, particularly in series where many cases have been treated conservatively. The mandible is the most common primary site (ratio mandible: maxilla 8:1), with no consistent differences related to sex or ethnicity [6]. The reported mean time from primary to diagnosis of metastasis varies from 9 to 18 years (14 years in this case) [4–6, 8–10].

Whilst metastasizing ameloblastomas are rare, the lungs are the most common site of distant spread [7]. In the present case, the original core biopsy of the pulmonary mass contained solid sheets of poorly differentiated epithelioid cells with areas of focal squamous differentiation which, alongside p40 staining, resulted in the erroneous diagnosis of squamous cell carcinoma. Upon retrospective review, there are features within the core biopsy to support a diagnosis of ameloblastoma with morphology consistent with the larger surgical specimen.

The original misdiagnosis resulted in, to our knowledge, the first case of a metastasizing ameloblastoma treated with neoadjuvant chemoimmunotherapy. Whilst large areas of the tumor exhibited the classical morphology of a conventional ameloblastoma, there were several small foci of necrosis and increased mitotic activity, neither of which have been reported in other cases (Fig. 3). The differential diagnosis of ameloblastic carcinoma was carefully considered, but as the only concerning feature was small foci of necrosis, and cytological features of malignancy were not identified, this diagnosis was not established. On these grounds, the small foci of necrosis and increased mitotic rate may be attributable to the effects of the neoadjuvant treatment.

Modalities for the treatment of metastasizing ameloblastoma have been reported to include surgery, chemotherapy and radiotherapy either in isolation or combination [7]. No treatment modality (or combination thereof) has been found to be superior with respect to either metastasizing ameloblastomas, or pulmonary metastasizing ameloblastomas more specifically [7, 11]. Indeed, the relative effectiveness of different treatment modalities is difficult to evaluate given the small number of cases of this rare entity. In this case the neoadjuvant treatment exerted minimal biological effect on the tumor largely in the form of focal necrosis, with most of the lesion remaining unaffected. This would suggest that this combination of neoadjuvant chemo/immunotherapy was of limited benefit in this case. A molecularly targeted approach has been proposed for ameloblastoma treatment as the BRAF V600E mutation has been found in 2/3 of cases of such tumors [12, 13]. They have proposed the use of BRAF-inhibitors in a neoadjuvant capacity in ameloblastoma with this molecular profile, but mutation of BRAFV600E was not identified here. In the present case we report a AKT1 c.49G > A p.(Glu17Lys) exon 4 variant, a hotspot mutation which is commonly associated with various cancers, including breast, colorectal and lung [14], but has also been recently identified in an ameloblastoma with a BRAF mutated background [15]. Other investigators have noted alterations in the AKT signaling pathway [16]. These observations may open the possibility of therapy with inhibitors of AKT1 signaling using therapeutics such as MK2206, as has been described in other cancers [17]. One recent report also described the use of the PI3K inhibitor Copanlisib in a patient with metastasizing ameloblastoma with a PI3K mutation (on a BRAF mutated background). This resulted in a durable partial response [13].

Precisely how and why some benign conventional ameloblastomas spread to distant sites remains unclear, and this is a source of considerable speculation in the literature [7]. Possible explanations for pulmonary deposits of ameloblastoma include hematogenous metastasis, aspiration of fragments of tumor during operative procedures or implantation during surgical manipulations and are reviewed elsewhere [6–8]. In our case, the six surgical episodes this patient underwent will have created many opportunities for aspiration and /or surgical implantation, as multiple recurrences and surgical interventions are commonly reported in the literature cohorts [6].

The present case serves to highlight the importance of considering the exceedingly rare metastasizing ameloblastoma in any patient with a history of an ameloblastoma and presenting with a pulmonary mass, particularly as the latter can mimic features of a squamous cell carcinoma. Whilst neoadjuvant chemo/immunotherapy appears to have been of limited, if any, benefit in this case, there is potential for novel targeted therapeutics [12].

## Supplementary Information

Below is the link to the electronic supplementary material.

Supplementary Material 1

## Data Availability

No datasets were generated or analysed during the current study.

## Code Availability

Not applicable: no code was used in this paper

## References

[1] WHO Classification of Tumors Editorial Board (2024) WHO classification of tumors: head and neck tumors, vol 9, 5th edn. The International Agency for Research on Cancer (IARC), Lyon

[2] Rizzitelli A, Smoll NR, Chae MP, Rozen WM, Hunter-Smith DJ (2015) Incidence and overall survival of malignant ameloblastoma. PLoS ONE 10(2):e0117789 25692490 10.1371/journal.pone.0117789 PMC4333213

[3] Sarode G, Gondivkar SM, Gore A, Anand R, Sengupta N, Mehta V et al (2023) Clinico-pathological and prognostic overview of metastasizing ameloblastoma: an overview of the systematic reviews. J Oral Biol Craniofac Res 13(6):751–757 38028232 10.1016/j.jobcr.2023.10.006 PMC10661192

[4] Chrcanovic BR, Martins-Chaves RR, Pontes FSC, Fonseca FP, Gomez RS, Pontes HAR (2022) Comparison of survival outcomes between ameloblastic carcinoma and metastasizing ameloblastoma: A systematic review. J Oral Pathol Med 51(7):603–610 35822408 10.1111/jop.13334 PMC9544829

[5] Slootweg PJ, Müller H (1984) Malignant ameloblastoma or ameloblastic carcinoma. oral surgery, oral medicine. Oral Pathol 57(2):168–176 10.1016/0030-4220(84)90207-x 6366686

[6] Van Dam SD, Unni KK, Keller EE (2010) Metastasizing (malignant) ameloblastoma: review of a unique histopathologic entity and report of Mayo clinic experience. J Oral Maxillofac Surg 68(12):2962–2974 20970910 10.1016/j.joms.2010.05.084

[7] Pandiar D, Anand R, Kamboj M, Narwal A, Shameena PM, Devi A (2021) Metastasizing ameloblastoma: A 10 year clinicopathological review with an insight into pathogenesis. Head Neck Pathol 15(3):967–974 33394372 10.1007/s12105-020-01258-5 PMC8384989

[8] Dissanayake RKG, Jayasooriya PR, Siriwardena DJL, Tilakaratne WM (2011) Review of metastasizing (malignant) ameloblastoma (METAM): pattern of metastasis and treatment. Oral Surg Oral Med Oral Pathol Oral Radiol Endod 111(6):734–741 21459020 10.1016/j.tripleo.2010.12.018

[9] Jayaraj G, Sherlin HJ, Ramani P, Premkumar P, Natesan A, Ramasubramanian A et al (2014) Metastasizing Ameloblastoma - a perennial pathological enigma? Report of a case and review of literature. J Craniomaxillofac Surg 42(6):772–779 24342734 10.1016/j.jcms.2013.11.009

[10] Laughlin EH (1989) Metastasizing ameloblastoma. Cancer 64(3):776–780 2663133 10.1002/1097-0142(19890801)64:3<776::aid-cncr2820640335>3.0.co;2-8

[11] Yang X, Zhou K, Tao Y, Ge S, Shang W, Song K (2022) Treatment efficacy and prognosis of pulmonary metastasizing ameloblastoma: a systematic review. Int J Oral Maxillofac Surg 51(5):579–590 34462177 10.1016/j.ijom.2021.07.016

[12] Ebeling M, Scheurer M, Sakkas A, Pietzka S, Schramm A, Wilde F (2023) BRAF inhibitors in BRAF V600E-mutated ameloblastoma: systematic review of rare cases in the literature. Med Oncol 40(6):163 37115331 10.1007/s12032-023-01993-z PMC10147738

[13] Lynch MM, Hermida-Viveiros P, Stencel S, Knott H, Al-Maryati R, Obeidin F et al (2025) Durable disease regression with Copanlisib treatment in PI3K-mutated metastasizing ameloblastoma: A case report. Rare Tumors. ;17 10.1177/20363613241309961 PMC11707780 39790867

[14] Chen Y, Huang L, Dong Y, Tao C, Zhang R, Shao H et al (2020) Effect of AKT1 (p. E17K) hotspot mutation on malignant tumorigenesis and prognosis. Front Cell Dev Biol. ;8 10.3389/fcell.2020.573599 PMC7573235 33123537

[15] Marín-Márquez C, Adisa AO, Niklander SE, Kirby J, Hunter KD (2025) Genomic and transcriptomic analysis of ameloblastoma reveals distinct molecularly aggressive phenotypes. Mod Pathol 38(3):100682 39675431 10.1016/j.modpat.2024.100682

[16] Cecim RL, Carmo HAF, Kataoka MSS, Freitas VM, de Melo Alves Júnior S, Pedreira EN et al (2014) Expression of molecules related to AKT pathway as putative regulators of ameloblastoma local invasiveness. J Oral Pathol Med 43(2):143–147 23837696 10.1111/jop.12103

[17] Zhao T, Tian Y, Ding X, Liu L, Tan B, Yang B et al (2021) Genetic analysis and targeted therapy using buparlisib and MK2206 in a patient with triple metachronous cancers of the kidney, prostate, and squamous cell carcinoma of the lung: A case report. Onco Targets Ther 14:2839–2845 33953569 10.2147/OTT.S298697 PMC8091866

