# Supplementary material for: Metastasizing Ameloblastoma Mimicking Squamous Cell Carcinoma of the Lung and Harboring an AKT1 Mutation
Source: Head Neck Pathol. 2025 Oct 27;19(1):120. doi: 10.1007/s12105-025-01844-5 (PMC12559519; doi:10.1007/s12105-025-01844-5)
Supplement: Supplementary file 1 — Supplementary Material 1 [file 12105_2025_1844_MOESM1_ESM.docx]

**Supplementary material**

**S1: Oncomine Precision assay gene list**

DNA Hotspots: *AKT1 CHEK2 FGFR3 KIT NTRK3 AKT2 CTNNB1 FGFR4 KRAS PDGFRA AKT3 EGFR FLT3 MAP2K1 PIK3CA ALK ERBB2 GNA11 MAP2K2 PTEN AR ERBB3 GNAQ MET RAF1 ARAF ERBB4 GNAS MTOR RET BRAF ESR1 HRAS NRAS ROS1 CDK4 FGFR1 IDH1 NTRK1 SMO CDKN2A FGFR2 IDH2 NTRK2 TP53*

*CNVs: ALK FGFR1 AR FGFR2 CD274 FGFR3 CDKN2A KRAS EGFR MET ERBB2 PIK3CA ERBB3 PTEN*

*Inter-genetic fusions: ALK NTRK1 BRAF NTRK2 ESR1 NTRK3 FGFR1 NUTM1 FGFR2 RET FGFR3 ROS1 MET RSPO2 NRG1 RSPO3*

*Intra genetic fusions: AR, EGFR, MET*
